# Diagnostic and Prognostic Value of hsa_piR_022710, hsa_piR_019822, and hsa_piR_020840 in Early-Stage Non-Small-Cell Lung Cancer: Implications for Recurrence and Survival in Squamous Cell Carcinoma Patients

**DOI:** 10.3390/ijms26072870

**Published:** 2025-03-21

**Authors:** Yangyi He, Antonio Altuna-Coy, Melissa Acosta-Plasencia, Laureano Molins, David Sánchez-Lorente, Daniel Martinez, Tania Díaz, Risha Na, Ramón M. Marrades, Alfons Navarro

**Affiliations:** 1 Molecular Oncology and Embryology Laboratory, Human Anatomy and Embryology Unit, Department of Surgery and Medical-Surgical Specialties, Faculty of Medicine and Health Sciences, Universitat de Barcelona (UB), c. Casanova 143, 08036 Barcelona, Spain; yangyihe@ub.edu (Y.H.); aaltunacoy@ub.edu (A.A.-C.); macostapl@ub.edu (M.A.-P.); tdiaz@ub.edu (T.D.); rishana@ub.edu (R.N.); 2 School of Basic Medical Sciences, Chengdu University, Chengdu 610106, China; 3 Department of Thoracic Surgery, Hospital Clínic de Barcelona, Universitat de Barcelona (UB), 08036 Barcelona, Spain; lmolins@clinic.cat; 4 Thoracic Oncology Unit, Hospital Clínic de Barcelona, Universitat de Barcelona (UB), 08036 Barcelona, Spain; dmartin1@clinic.cat (D.M.); marrades@clinic.cat (R.M.M.); 5 Institut d’Investigacions Biomèdiques August Pi i Sunyer (IDIBAPS), c. Villarroel 170, 08036 Barcelona, Spain; 6 Department of Thoracic Surgery, Parc Taulí Hospital Universitari, Institut d’Investigació i Innovació Parc Taulí (I3PT), Universitat Autònoma de Barcelona, 08202 Sabadell, Spain; dsanchezl@tauli.cat; 7 Department of Pathology, Hospital Clínic de Barcelona, Universitat de Barcelona (UB), 08036 Barcelona, Spain; 8 Department of Pneumology, Institut Clínic Respiratori (ICR), Hospital Clínic de Barcelona, Universitat de Barcelona (UB), 08036 Barcelona, Spain; 9 Centro de Investigación Biomédica en Red de Enfermedades Respiratorias (CIBERES), Instituto de Salud Carlos III, 28029 Madrid, Spain

**Keywords:** NSCLC, squamous cell carcinoma, piRNA, recurrence, prognosis, biomarkers

## Abstract

Despite significant advancements in early detection and treatment, non-small-cell lung cancer (NSCLC) remains a leading cause of cancer-related mortality. Specifically, in early-stage cases, recurrence after surgery continues to be the principal cause of death for these patients. The urgent need for novel diagnostic and prognostic biomarkers has directed attention towards PIWI-interacting RNAs (piRNAs), a group of small RNAs that regulate genomic stability and epigenetics. Some piRNAs, including hsa_piR_022710, hsa_piR_019822, and hsa_piR_020840, have been described as deregulated in various cancers. This study investigated the expression of these three piRNAs by RT-qPCR in 277 NSCLC patients and developed survival and CART classification models to predict recurrence risk, overall survival (OS), and disease-free survival (DFS). hsa_piR_019822 and hsa_piR_020840 were able to discriminate between tumor and normal tissue, as well as between adenocarcinoma and squamous cell carcinoma (LUSC) patients. Elevated expression of hsa_piR_019822 and hsa_piR_022710 was correlated with an increased risk of recurrence and poorer DFS and OS in LUSC patients. Patients with high hsa_piR_022710 expression more greatly benefited from adjuvant treatment. In summary, higher piRNA levels were associated with an increased risk of recurrence and poorer survival outcomes, especially in LUSC patients, where they may help guide personalized treatment strategies.

## 1. Introduction

Lung cancer, with approximately 2.5 million new cases and over 1.8 million fatalities globally, emerged as the primary cause of cancer-related morbidity and mortality in 2022 [1]. Its high lethality is largely attributed to late-stage diagnoses and limited effective therapeutic options for advanced disease [2]. Even for patients diagnosed at earlier stages who undergo surgical resection as the primary treatment, survival rates remain low due to the high risk of tumor recurrence [3,4]. NSCLC accounts for approximately 85% of all cases, making it the predominant form of this highly aggressive disease [5]. Histological classification of NSCLC includes several subtypes, but the two most frequent types are lung adenocarcinoma (LUAD) and LUSC, which differ in their cellular origin, molecular characteristics, and associated risk factors [6]. LUAD, the most common subtype (45%), originates in the glandular cells of the lung and is characterized by mutations in key driver genes, including *EGFR*, *KRAS*, and *ALK*, which have paved the way for targeted therapies [7]. In contrast, LUSC (25–30%) arises from the squamous epithelial cells and is strongly associated with a history of smoking [8,9]. Due to its high degree of molecular complexity, LUSC lacks targetable genetic abnormalities [10].

In the case of early-stage patients, with both LUAD and LUSC, postoperative recurrence occurs in nearly half of the patients within five years, and recurrent disease is often more aggressive and less responsive to treatment [11]. The persistent challenges of recurrence and metastasis underscore the urgent need for novel biomarkers to guide clinical decision-making after surgery [12,13]. Current prognostic tools rely heavily on traditional clinical and pathological parameters, such as tumor size, stage, and histological subtype [14]. However, these factors often fail to capture the underlying molecular mechanisms that drive tumor recurrence and progression [15]. Identifying reliable molecular biomarkers could provide deeper insights into these mechanisms, enable risk stratification of patients, and facilitate the development of precision treatment strategies to improve patient outcomes.

In recent years, non-coding RNAs (ncRNAs) have been recognized as critical regulators of various biological functions across different cell types and tissues [16]. Their dysregulation has been linked to numerous diseases, making them pivotal players in cancer biology, and offering new insights into tumorigenesis, progression, and metastasis [17]. Among these molecules, piRNAs are a group of small non-coding RNAs ranging in size from 24 to 30 nucleotides discovered in germline cells, where they are preferentially expressed [18]. The family of piRNAs includes more than 23,000 members according to piRNAbank and piRBase [19]. They bind to PIWI family proteins and have several functions including epigenetic regulation [20]. piRNAs have been recently detected in cancer cells exhibiting diverse regulatory roles. They are well known for their roles in maintaining genomic stability and silencing transposable elements [21]. Unlike many other small ncRNAs, they function without relying on the Dicer enzyme. Instead, they bind to the PIWI subfamily of Argonaute proteins, which are essential for maintaining genomic stability in germ cells [22]. The growing evidence positions piRNAs as highly promising biomarkers for NSCLC and other cancers capable of improving prognostic accuracy and guiding precision treatment strategies [23,24,25,26]. In the present study, we selected some piRNAs of interest, hsa_piR_020840, hsa_piR_019822, and hsa_piR_022710, that have been previously described for their role in the carcinogenesis process in other tumor types. hsa_piR_020840 was significantly overexpressed in gastric cancer tissues compared to healthy tissues [27]; hsa_piR_019822 was detected in 11 lung cancer cell lines of different histological types [28]; and hsa_piR_022710 has been identified as a key piRNA enriched in neuroblastoma, with its associated target genes playing critical roles in tumor-related events, and potentially in tumor microenvironment remodeling [29]. The three piRNAs have been associated with key tumor-related events such as cell proliferation, immune evasion, and tumor microenvironment remodeling. Moreover, their overexpression highlights their potential use as biomarkers for diagnosis, prognosis, and therapeutic response assessment. Therefore, we decided to analyze their expression levels in our cohort of NSCLC patients and study their potential impact on patient outcomes.

## 2. Results

### 2.1. Clinical and Pathological Characteristics of Patients

**Figure 1:**
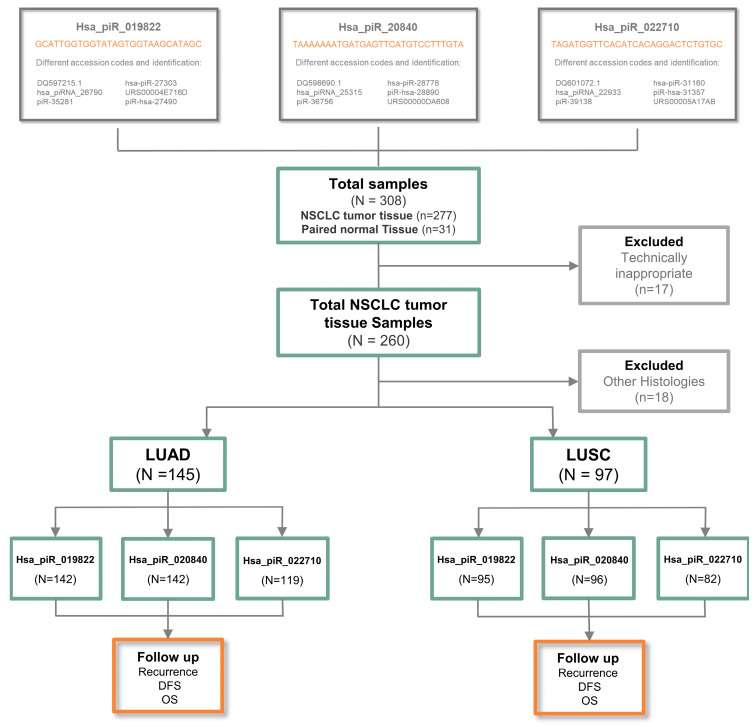
Information about hsa_piR_019822, hsa_piR_020840, and hsa_piR_022710 sequences and REMARK diagram of the study. DFS, disease-free survival; OS, overall survival.

The final study consisted of 260 resected lung cancer patients diagnosed with NSCLC (Figure 1). Focusing on their demographic and clinical characteristics (Table 1), 191 (73.5%) were male; the average age of patients was 67 years, with 58.1% being older than 65 years; and 233 (88.9%) of the patients had a smoking-related history, with 37.3% being current smokers and 51.6% being former smokers. TNM staging revealed that stage I was the most common (60.4% of cases). Most patients did not receive adjuvant therapy after resection surgery (69.4%). Relapse occurred in 40.8% of cases. The average follow-up time was 61 months, providing a robust basis for assessing long-term outcomes.

In the LUAD cohort, 61.4% were male, while the LUSC cohort showed a slightly higher male predominance (90.7%). The average age was 66 years in the LUAD cohort and 68 years in the LUSC cohort. In the LUSC cohort, all patients were current or former smokers. In contrast, in the LUAD cohort, this proportion was 81.4%. This highlights a stronger smoking association in LUSC patients [30].

LUAD patients were more frequently diagnosed at stage I (62.8%) compared to LUSC patients (56.7%). These findings suggest that LUAD patients tend to be diagnosed at earlier stages, while LUSC patients are more likely to be diagnosed at relatively later stages, potentially reflecting differences in tumor progression or detection patterns [30]. Relapse occurred at a higher rate in LUAD (44.8%) than in LUSC (33%).

### 2.2. Patient Prognostic Predictors

We analyzed all patients’ recurrence status, Time to Relapse (TTR), DFS, survival status, and OS at 1 year, 2 years, 3 years, and 5 years.

In the LUAD cohort, recurrence emerged as the most critical determinant of the survival outcome, significantly influencing each interval (*p* < 0.001). Tumor size, lymph node status, and disease stage also played pivotal roles. Tumor size had a significant impact on DFS and TTR at 1, 2, and 3 years (*p* < 0.005), while lymph node involvement consistently affected these metrics across all intervals, with the strongest influence observed at 2 and 3 years (*p* < 0.001). Disease stage was a consistent determinant for DFS, TTR, and OS throughout the study period (*p* < 0.05). Furthermore, patients with emphysema have a poor prognosis for long-term OS outcomes (Supplementary Table S1).

In the LUSC cohort, survival outcomes were primarily determined by relapse (*p* < 0.001). Other factors, including tumor size, lymph node status, and disease stage, showed no significant influence. TTR, DFS, and OS were not significantly associated with these factors at any interval.

### 2.3. PiRNAs Expression in Patient Sample

**Figure 2:**
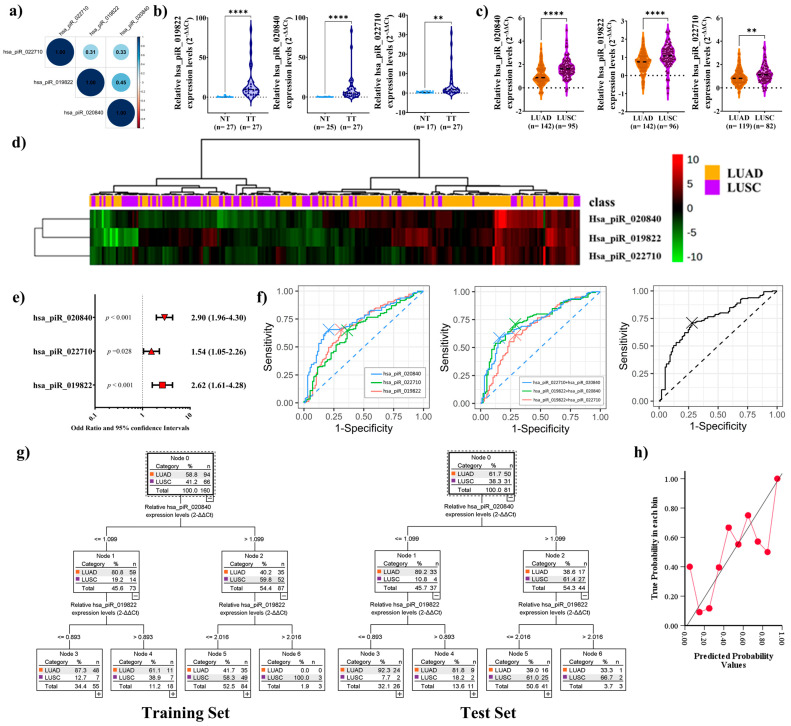
(**a**) Spearman correlation coefficients were calculated to construct a correlation matrix for the expression of three piRNAs: hsa_piR_022710, hsa_piR_019822, and hsa_piR_020840. Positive correlations are represented by graded blue colors, while correlations with *p*-values ≥ 0.05 are considered insignificant and left blank. The intensity of the color and the size of the circles are proportional to the correlation coefficients. The color legend on the right side of the correlogram indicates the correlation coefficients and their corresponding colors. (**b**) Violin plot analysis of expression of three piRNAs, hsa_piR_019822, hsa_piR_020840, and hsa_piR_022710, in tumor and paired normal tissue or (**c**) LUAD and LUSC. Each violin plot shows the median, quartiles, and extreme values. (**d**) Heatmap of the clustering analysis. Patients were categorized as LUAD or LUSC. piRNA expression intensities are displayed as colors ranging from red grading (more abundant) to green grading (less abundant). (**e**) Odds ratio (OR values in different red shapes for each parameter) and 95% confidence intervals. Dotted vertical line: An OR greater than 1 indicates that high expression levels of the piRNA are likely to occur in LUSC patients. An OR of less than 1 indicates that high expression levels of the piRNA are likely to occur in LUAD patients. (**f**) Receiver operating characteristic (ROC) comparison with the independent, paired, and combination expression of three piRNAs. (**g**) Classification and Regression Decision Tree (CART) model for lung cancer subtype diagnosis (Training Set and Test Set) by the combination of hsa_piR_019822 + hsa_piR_022710. (**h**) Calibration plot assessing the alignment between predicted probabilities and observed outcomes. The *X*-axis represents predicted probabilities, and the *Y*-axis represents observed probabilities. The solid line indicates perfect calibration, while the red line connects points grouped by probability bins, illustrating the model’s actual calibration. ** *p* < 0.01; **** *p* < 0.0001. Abbreviations: NT, normal tissue; TT, tumoral tissue; LUAD, lung adenocarcinoma; LUSC, lung squamous cell carcinoma.

We analyzed the expression of the three piRNAs in tumor tissues and their corresponding adjacent normal tissues from patients diagnosed with NSCLC subtypes LUAD and LUSC. The correlations matrix for the expression of the three piRNAs, hsa_piR_022710, hsa_piR_019822, and hsa_piR_020840, indicated that each pair of piRNAs showed a positive correlation (Figure 2a).

Moreover, a significant upregulation of the three piRNAs was observed in tumor tissues (*p* < 0.001, *p* < 0.001, and *p* = 0.004, respectively) (Figure 2b). Interestingly, the three piRNAs were significantly upregulated in LUSC patients compared to those with LUAD (*p* < 0.001, *p* < 0.001, *p* = 0.002, Figure 2c). When the samples were grouped by hierarchical clustering based on their expression profiles using the MetaboanalystR package in R, the heatmap revealed distinct clustering patterns aligned with the classifications, indicating that the expression levels of these piRNAs could differentiate between LUAD and LUSC (Figure 2d).

Log OR analysis was conducted to study whether the above-mentioned piRNAs could be used as an independent predictor to distinguish both lung cancer subtypes. The results confirmed that the higher expression of hsa_piR_022710, hsa_piR_019822, and hsa_piR_020840 could indicate the presence of LUSC (*p* < 0.001, *p* < 0.001, *p* = 0.028, Figure 2e). The receiver operating characteristic (ROC) analysis evaluated the diagnostic performance of three piRNAs and their combinations in distinguishing LUAD from LUSC. Among the individual piRNAs, hsa_piR_020840 showed the best diagnostic performance [area under the curve (AUC): 0.732, sensitivity: 65.5%, specificity: 79.4%]. The combination of hsa_piR_019822 + hsa_piR_020840 achieved the highest overall accuracy, improving both sensitivity and specificity (AUC: 0.744, sensitivity: 71.0%, specificity: 71.1%). Combining all three piRNAs also resulted in high diagnostic accuracy, comparable to the best-performing combination (AUC: 0.742, sensitivity: 71.0%, specificity: 72.2%) (Figure 2f).

Classification and Regression Tree (CART) analysis was employed to develop a predictive model for distinguishing LUAD and LUSC based on hsa_piR_020840 and hsa_piR_019822 relative expression levels since it is the best model according to AUC analysis. Cross-validation confirmed the robustness of the model, which, with the remaining 30 percent of the cohort, correctly classified 27 of 31 LUSC patients, and 33 of 50 LUAD patients were correctly classified by the model in the Test Set (Figure 2g).

The calibration plot was created to evaluate the performance of the predictive model by comparing predicted probabilities with actual outcomes. In this case, the model is undercalibrated at low probabilities (0.0–0.4), underestimating the actual outcomes as the points fall below the diagonal. At medium probabilities (0.4–0.8), the model improves, aligning more closely with the reference line. Finally, at high probabilities (0.8–1.0), the model shows good calibration, reflecting more accurate predictions. Overall, the model is reliable at intermediate and high probabilities (Figure 2h).

These findings suggest the potential biomarker roles for hsa_piR_020840 and hsa_piR_019822 in distinguishing both lung cancer subtypes.

### 2.4. Elevated piRNA Expression Levels Correlate with Higher Recurrence Risk in LUSC Patients

Since significant differences in piRNA expression levels were observed between LUAD patients (n = 145) and LUSC patients (n = 97), we decided to evaluate the prognostic role of piRNAs separately within each histological subtype, focusing on critical outcomes such as recurrence, survival status, and disease presence. To achieve this, we established two time points for analysis: short term (2 years) and long term (5 years). This approach aimed to better understand the unique contributions of piRNAs, enabling improved risk stratification and personalized management strategies. The methodology and workflow are outlined in the REMARK diagram in Figure 1.

**Figure 3:**
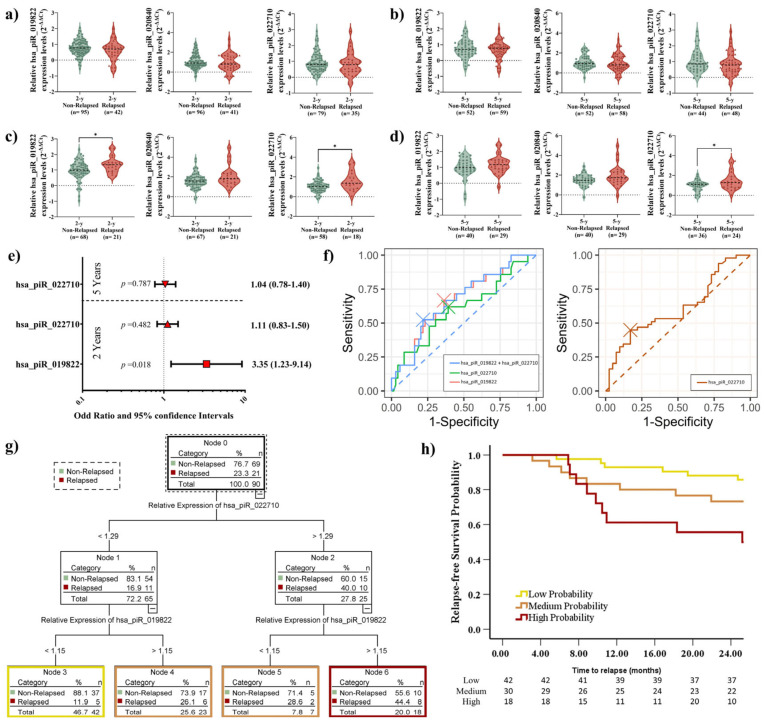
(**a**) Violin plot analysis of expression of three piRNAs, hsa_piR_019822, hsa_piR_020840, and hsa_piR_022710, in LUAD for the short-term outcome and (**b**) long-term outcome regarding recurrence. The thick dotted line within each violin represents the median, while the thinner dotted lines indicate the interquartile range (IQR). The horizontal dotted line at 0 represents the baseline expression level for comparison. (**c**) Violin plot analysis of expression of three piRNAs, hsa_piR_019822, hsa_piR_020840, and hsa_piR_022710, in LUSC for the short-term outcome and (**d**) long-term outcome regarding recurrence. The thick dotted line within each violin represents the median, while the thinner dotted lines indicate the interquartile range (IQR). The horizontal dotted line at 0 represents the baseline expression level for comparison. (**e**) Odds ratio (OR values in different red shapes for each parameter) and 95% confidence intervals. Dotted vertical line: An OR greater than 1 indicates that high expression levels of the piRNAs are likely to occur in relapsed LUSC patients. An OR of less than 1 indicates that high expression levels of the piRNAs are likely to occur in non-relapsed LUSC patients. (**f**) Receiver operating characteristic (ROC) comparison with the independent and paired expression of two piRNAs. (**g**) Classification and Regression Decision Tree (CART) model for recurrence prognosis by the combination of hsa_piR_019822 + hsa_piR_022710. (**h**) Kaplan–Meier plot of the recurrence of LUSC cases stratified by the cutoff value of hsa_piR_019822 + hsa_piR_022710 (high, medium, and low probability). * *p* < 0.05.

In LUAD patients, no significant differences in recurrence status were observed at any time point during the follow-up period based on piRNA expression levels (Figure 3a,b). Interestingly, we observed significant differences in hsa_piR_019822 (*p* = 0.023) and hsa_piR_022710 (*p* = 0.02) expression levels in relation to relapse in the short-term outcome (Figure 3c). Moreover, a difference in hsa_piR_022710 expression in the long-term outcome was observed (*p* = 0.05) (Figure 3d). OR analysis confirmed only the potential role of hsa_piR_019822 as an independent predictor in short-term relapse outcomes (*p* = 0.018, Figure 3e).

Among individual piRNAs, hsa_piR_019822 demonstrated the highest diagnostic accuracy (AUC: 0.669, sensitivity: 66.7%, specificity: 63.8%). The combination of hsa_piR_019822 + hsa_piR_022710 achieved the highest accuracy at the 2-year follow-up (AUC: 0.669, sensitivity: 52.4%, specificity: 78.3%) (Figure 3f). The AUC of hsa_piR_022710 for the 5-year follow-up (AUC: 0.612, sensitivity: 44.9%, specificity: 82.9%) was not good enough to distinguish relapse in the long-term outcome (Figure 3f).

We determined the optimal cutoff value for hsa_piR_019822 and hsa_piR_022710 using X-tile software, which allowed us to stratify patients into distinct groups based on their piRNA expression levels. Cutoff values were hsa_piR_019822: 1.15 and hsa_piR_022710: 1.29.

CART analysis was employed to develop a predictive model for stratifying high-risk recurrence based on the cutoff values of the relative expression levels of hsa_piR_019822 and hsa_piR_022710 using X-tile. Due to higher expression of both piRNAs being associated with a higher recurrence likelihood (Figure 3c), patients were stratified: hsa_piR_019822 levels > 1.15 and hsa_piR_022710 > 1.29 were classified as high probability (n = 18), hsa_piR_019822 levels > 1.15 and hsa_piR_022710 ≤ 1.29 or hsa_piR_019822 levels ≤ 1.15 and hsa_piR_022710 > 1.29 were classified as medium probability (n = 30), and hsa_piR_019822 levels ≤ 1.15 and hsa_piR_022710 ≤ 1.29 were low probability (n = 42) (Figure 3g). Kaplan–Meier analysis revealed significant differences in recurrence status between patients with low and high recurrence probabilities after 2 years of follow-up (*p* = 0.003). Specifically, patients with higher expression levels of both piRNAs were found to have a 2.2-fold higher probability of relapse compared to those with lower piRNA expression levels. However, no significant differences were observed in recurrence status for medium-probability patients when compared to either low-probability (*p* = 0.099) or high-probability (*p* = 0.232) patients. These findings suggest that piRNA expression levels may play a more decisive role in distinguishing patients at the extremes of recurrence risk, while medium-probability patients require further investigation to determine their relapse patterns (Figure 3h).

### 2.5. Elevated piRNA Expression Levels Correlate with Poor DFS in LUSC Patients During Short-Term Follow-Up

**Figure 4:**
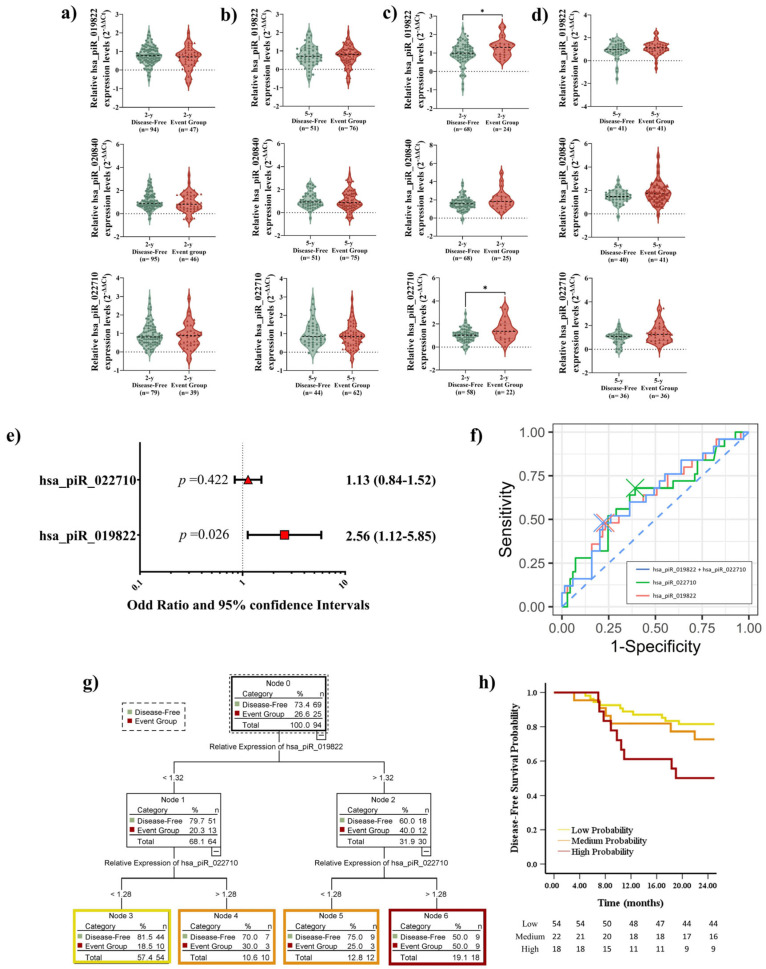
(**a**) Violin plot analysis of the expression of three piRNAs, hsa_piR_019822, hsa_piR_020840, and hsa_piR_022710, in LUAD for short-term outcomes and (**b**) long-term outcomes regarding DFS. The thick dotted line within each violin represents the median, while the thinner dotted lines indicate the interquartile range (IQR). The horizontal dotted line at 0 represents the baseline expression level for comparison. (**c**) Violin plot analysis of the expression of three piRNAs, hsa_piR_019822, hsa_piR_020840, and hsa_piR_022710, in LUSC for short-term outcomes and (**d**) long-term outcomes regarding DFS. The thick dotted line within each violin represents the median, while the thinner dotted lines indicate the interquartile range (IQR). The horizontal dotted line at 0 represents the baseline expression level for comparison. (**e**) Odds ratio (OR) values (shown in different red shapes for each parameter) and 95% confidence intervals. The dotted vertical line indicates that an OR greater than 1 suggests that high expression levels of the piRNAs are likely to occur in the event group of LUSC patients, while an OR of less than 1 suggests that high expression levels of the piRNAs are likely to occur in disease-free LUSC patients. (**f**) Receiver operating characteristic (ROC) comparison of the independent and paired expression of two piRNAs. (**g**) Classification and Regression Decision Tree (CART) model for DFS prognosis by the combination of hsa_piR_019822 + hsa_piR_022710. (**h**) Kaplan–Meier plot of DFS in LUSC cases stratified by the cutoff value of hsa_piR_019822 + hsa_piR_022710 (high, medium, and low probability). * *p* < 0.05.

Following the same approach as the above analysis, we measured the expression levels of the piRNAs according to DFS. No significant differences in piRNA expression levels were observed at any time point during the follow-up period in LUAD patients (Figure 4a,b). In the LUSC cohort, the expression levels of hsa_piR_019822 (*p* = 0.0341) and hsa_piR_022710 (*p* = 0.0326) showed significant differences in short-term outcomes (Figure 4c). However, we did not find significant differences in analyzing long-term outcomes (Figure 4d). OR analysis confirmed that hsa_piR_019822 has the potential to serve as an independent predictor for short-term outcomes (*p* = 0.026, Figure 4e). The combination of hsa_piR_019822 + hsa_piR_022710 achieved the highest accuracy at the 2-year follow-up (AUC: 0.632, sensitivity: 48.0%, specificity: 78.3%). Considering each individual piRNA, hsa_piR_019822 demonstrated the same diagnostic accuracy (AUC: 0.627, sensitivity: 48.0%, specificity: 76.8%) as hsa_piR_022710 (AUC: 0.627, sensitivity: 68.0%, specificity: 60.9%) (Figure 4f).

We determined the optimal cutoff value for hsa_piR_019822 (1.32) and hsa_piR_022710 (1.28) using the X-tile software.

CART analysis was applied to develop a predictive model based on the relative expression levels of hsa_piR_019822 and hsa_piR_022710. Due to the association between higher expression levels of the two piRNAs and an increased likelihood of disease progression, patients were categorized into the following three risk groups: the high-probability group (n = 18), medium-probability group (n = 22), and low-probability group (n = 54) (Figure 4g). Kaplan–Meier analysis showed a significant difference in disease status between the low- and high-event-rate groups after a 2-year follow-up (*p* = 0.007). Specifically, patients in the high-probability group were 2.7 times more likely to experience recurrence compared to those in the low-probability group. However, when comparing the medium-probability group with the other two groups, no significant differences were observed (*p* = 0.159 with high probability, *p* = 0.397 with low probability). These results suggest that piRNA expression levels may play a role in distinguishing DFS risk (Figure 4h).

### 2.6. Elevated piRNA Expression Levels Correlate with Poor OS in LUSC Patients During Short-Term Follow-Up

**Figure 5:**
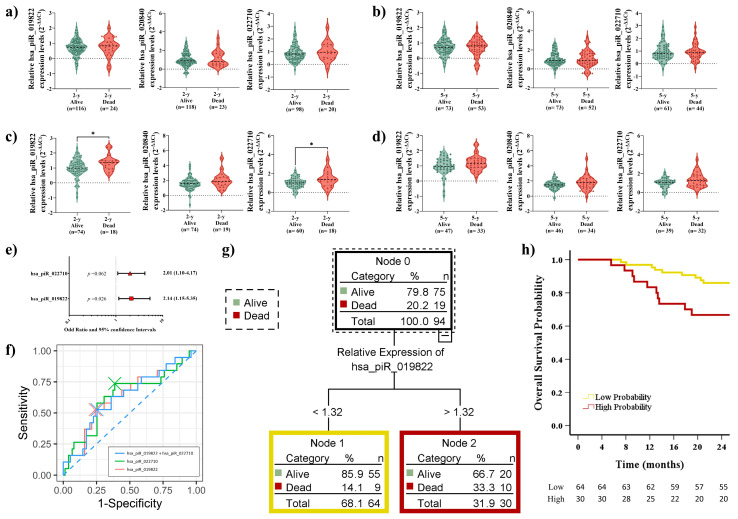
(**a**) Violin plot analysis of expression of three piRNAs, hsa_piR_019822, hsa_piR_020840, and hsa_piR_022710, in LUAD for short-term outcome and (**b**) long-term outcome regarding OS. The thick dotted line within each violin represents the median, while the thinner dotted lines indicate the interquartile range (IQR). The horizontal dotted line at 0 represents the baseline expression level for comparison. (**c**) Violin plot analysis of expression of three piRNAs, hsa_piR_019822, hsa_piR_020840, and hsa_piR_022710, in LUSC for short-term outcome and (**d**) long-term outcome regarding OS. The thick dotted line within each violin represents the median, while the thinner dotted lines indicate the interquartile range (IQR). The horizontal dotted line at 0 represents the baseline expression level for comparison. (**e**) Odds ratio (OR values in different red shapes for each parameter) and 95% confidence intervals. Dotted vertical line: An OR greater than 1 indicates that high expression levels of the piRNAs are likely to occur in dead LUSC patients. An OR of less than 1 indicates that high expression levels of the piRNAs are likely to occur in alive LUSC patients. (**f**) Receiver operating characteristic (ROC) comparison with the independent and paired expression of two piRNAs. (**g**) Classification and Regression Decision Tree (CART) model for DFS prognosis by the expression of hsa_piR_019822. (**h**) Kaplan–Meier plot of the OS of LUSC cases stratified by the cutoff value of hsa_piR_019822 (high and low probability). * *p* < 0.05.

During the follow-up period, piRNA expression levels in LUAD patients consistently showed no significant differences related to OS (Figure 5a,b). In LUSC patients, the expression levels of hsa_piR_019822 (*p* = 0.026) and hsa_piR_022710 (*p* = 0.038) showed significant differences in short-term survival outcomes (Figure 5c). However, no significant differences were observed in long-term outcomes (Figure 5d). hsa_piR_019822 was confirmed through OR analysis to have potential as an independent predictor for short-term survival (*p* = 0.026, Figure 5e). Moreover, it demonstrated the highest diagnostic accuracy (AUC: 0.641, sensitivity: 52.6%, specificity: 76.0%). The combination of the two piRNAs did not yield a significant improvement (AUC: 0.631, sensitivity: 52.6%, specificity: 74.7%) (Figure 5f).

Since only hsa_piR_019822 was directly associated with OS, we calculated the cutoff value using X-tile (1.32) and classified the patients according to high hsa_piR_019822 expression (>1.32) and low hsa_piR_019822 expression (≤1.32). Using CART analysis, we developed two risk groups based on hsa_piR_019822 expression: the high-probability group (n = 30) and the low-probability group (n = 64) (Figure 5g). Kaplan–Meier analysis was performed to evaluate survival differences between the high- and low-probability groups. A significant difference in OS status between groups was observed (*p* = 0.021, Figure 5h). This indicates that patients with high expression levels of hsa_piR_019822 had worse survival outcomes (2.37 times).

### 2.7. Prognostic Impact of piRNA Expression Levels on Recurrence, Survival, and the Role of Adjuvant Treatment

Given that adjuvant treatment has been shown to improve patient prognosis [31], we aimed to determine whether the aforementioned piRNAs in this study can serve as biomarkers for monitoring the therapeutic process. We divided all patients into four to six groups based on adjuvant treatment after surgery and the corresponding expression levels of the selected piRNAs, as previously described regarding recurrence, DFS, and OS status.

**Figure 6:**
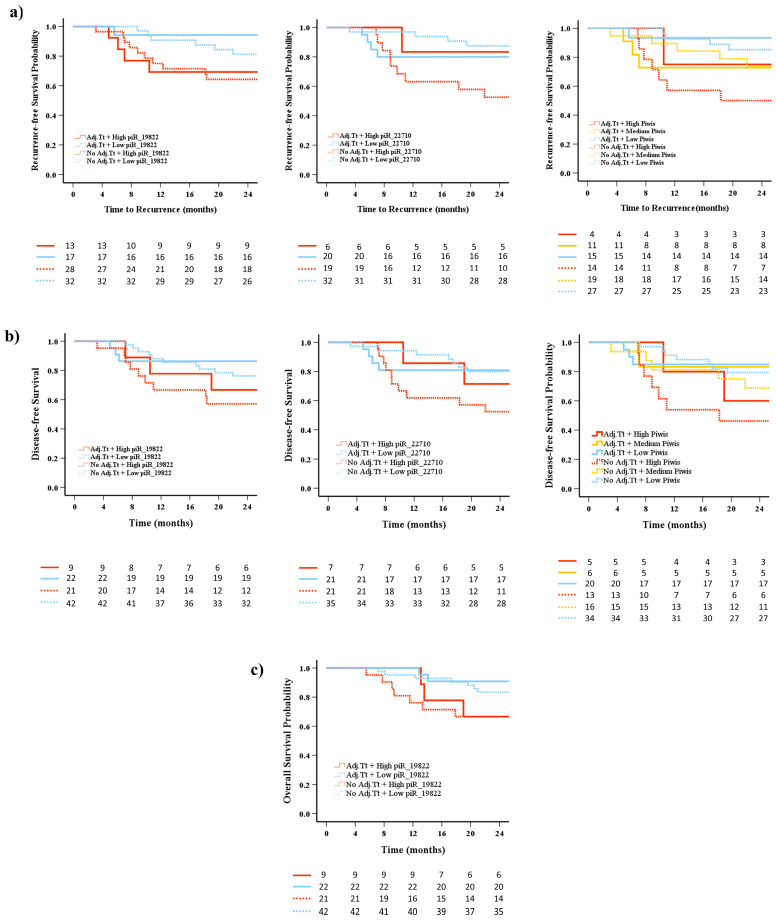
(**a**) Kaplan–Meier plot of the recurrence of LUSC cases stratified by the cutoff value of the combination of the adjuvant treatment with hsa_piR_019822 and/or hsa_piR_022710 (high, medium, and low expressions) for the short-term outcome. (**b**) Kaplan–Meier plot of the DFS of LUSC cases stratified by the cutoff value of the combination of the adjuvant treatment with hsa_piR_019822 and/or hsa_piR_022710 (high, medium, and low expressions) for the short-term outcome. (**c**) Kaplan–Meier plot of the DFS of LUSC cases stratified by the cutoff value of the combination of the adjuvant treatment with hsa_piR_019822 (high and low expression) for the short-term outcome.

Kaplan–Meier survival curves illustrated that patients with high hsa_piR_22710 expression could benefit from adjuvant treatment, as no differences were observed compared to patients with low expression who received treatment (*p* = 0.793), whereas differences were observed when they did not receive treatment (*p* = 0.004) (Figure 6a). No significant results were observed regarding hsa_piR_019822 (Figure 6a). The combination of the two piRNAs also showed that patients who received adjuvant treatment had no survival differences compared to those with low, medium, and high probability who did not receive adjuvant treatment (*p* = 0.468, *p* = 0.849, and *p* = 0.379, respectively) (Figure 6a).

Similar results were observed in the DFS analysis. Patients with high hsa_piR_22710 expression can benefit from adjuvant treatment, as no differences were observed compared to patients with low expression who received treatment (*p* = 0.720), whereas differences were observed when they did not receive treatment (*p* = 0.021) (Figure 6b).

In the OS analysis, we focused on hsa_piR_019822 since it was associated with OS, but no significant differences were observed (Figure 6c).

## 3. Discussion

Lung cancer recurrence rates increase with disease stage, ranging from 26 to 45% in stage I, 42 to 62% in stage II, and 70 to 77% in stage III [32,33,34]. In our cohort, the 40.8% recurrence rate reflects the high proportion of stage I diagnoses. Recurrence after complete resection is typically assessed via radiological methods. Since the discovery of EGFR mutations, it has significantly improved DFS in stage IB-IIIA EGFR-mutant NSCLC by targeting key mutations and reducing CNS recurrence [35], while analysis of other mutations (ALK, HER2, BRAF, NUTM1, IGF, KRAS) further optimizes treatment strategies [36,37,38,39]. However, these advancements are largely applicable to LUAD or non-LUSC patients, with limited relevance for those with LUSC.

The search for novel biomarkers has highlighted ncRNAs, particularly piRNAs, which regulate gene expression and genomic stability. Their dysregulation is linked to tumor progression in various cancers [40,41]. This study investigated the expression and prognostic significance of hsa_piR_022710, hsa_piR_019822, and hsa_piR_020840 in lung cancer. The results showed that the aforementioned piRNAs were significantly upregulated in tumor tissues compared to normal tissues, with higher expression in LUSC than in LUAD. We acknowledge that statistical significance does not always imply biological relevance, particularly in large cohorts. While the fold change between LUAD and LUSC appears modest (FC), small variations in piRNAs can have biological implications. Statistical significance reflects a consistent pattern, suggesting a role for piRNA biomarkers in distinguishing LUAD from LUSC.

The upregulation of these piRNAs in tumor tissues indicates their potential involvement in cancer development. Patients with elevated piRNA levels, especially elevated levels of hsa_piR_019822 and hsa_piR_022710, had a higher risk of recurrence and poorer survival outcomes, particularly in the short term. The stronger association observed in LUSC compared to LUAD suggests that piRNA expression patterns may vary based on tumor histology. This aligns with previous findings that LUSC and LUAD have distinct molecular characteristics [42], which may influence how piRNAs contribute to disease progression. In a previous study conducted by our research group, we showed that the PIWIL1 protein, which is involved in the biogenesis of piRNAs, becomes reactivated in the tumor tissues of some NSCLC patients. This reactivation was associated with worse patient outcomes [26,43]. Moreover, as we show in Supplementary Table S1, adjuvant treatment has a positive impact on relapse and survival status. Therefore, the lack of association between piRNA levels in LUAD regarding relapse and overall survival status may be due to the effect of adjuvant treatment on patient outcomes.

The CART model effectively stratified patients into different risk groups, suggesting that piRNAs could help identify individuals at higher risk of recurrence. Kaplan–Meier analysis confirmed that patients with high piRNA expression had significantly poorer survival.

Additionally, the expression of hsa_piR_022710 was associated with a better response to adjuvant therapy, suggesting that piRNA profiling could help guide postoperative treatment decisions. Although the effectiveness of adjuvant treatment for stage I NSCLC patients remains debated [44,45], Hsiao et al. demonstrated that platinum-based adjuvant chemotherapy improved survival in patients with surgically resected early-stage SCC [46]. By integrating our piRNA model, it may be possible to improve the prognosis of nearly 50% of patients with high expression levels by a factor of 2.84. Therefore, for patients not receiving adjuvant therapy, monitoring hsa_piR_022710 expression levels could be crucial. Patients with high expression of hsa_piR_022710 may require closer follow-up and monitoring to detect signs of early recurrence.

The role of piRNAs in lung cancer has not been extensively studied, leaving their potential functions and mechanisms largely unexplored. One possible mechanism underlying the role of piRNAs in lung cancer involves their interaction with tumor suppressor genes. Interestingly, based on the piRNAdb database, hsa_piR_019822 overlaps with different transcripts of LIMA1, indicating a potential regulatory interaction. LIMA1, also known as EPLIN (Epithelial Protein Lost In Neoplasm), is an actin-binding protein mainly found in epithelial tissues [47]. Its expression is often reduced in various cancers, including lung cancer [48]. Lower levels of EPLIN-α are linked to advanced disease stages and poorer prognosis, suggesting that LIMA1 may help suppress tumor cell growth and migration [49]. This suggests that hsa_piR_019822 may influence LIMA1 expression or function, potentially contributing to lung cancer progression. The other two piRNAs, hsa_piR_022710 and hsa_piR_020840, are also closely associated with multiple genes, but their specific mechanisms remain unreported. Further studies are needed to clarify the mechanisms underlying this interaction and its implications for tumor development. Similar associations between piRNA expression and tumor progression have been reported in other cancers. Our previous research, along with studies from other teams, has shown that piR-651 is also upregulated in NSCLC tissues and plays a role in promoting cell proliferation [43], inhibiting apoptosis, and regulating cell migration. Other examples are piR-823 and piR-932, which have been identified as being upregulated in breast cancer stem cells [50,51].

## 4. Materials and Methods

### 4.1. Patient Sample

We analyzed 277 tumor and 30 paired normal tissue samples from 277 adult patients diagnosed with NSCLC who underwent complete surgical resection at the Hospital Clinic of Barcelona from May 2007 to October 2019 (Figure 1). After collection, we immersed the samples in RNALater^®^ solution (ThermoFisher Scientific, Waltham, MA, USA) within 24 h. Subsequently, the tissues were sectioned into small pieces and stored at –80 °C until further processing. None of the patients included in the study received neoadjuvant therapy prior to surgery.

After the exclusion of the technically inappropriate (issues with sample quality, insufficient sensitivity of detection methods), the final 260 patients were included in our study cohort, and were stratified by histology: as LUAD (n = 145) or LUSC (n = 97) (Figure 1). Characteristics of the patients are detailed in Table 1.

### 4.2. RNA Extraction and piRNA Quantification

Total RNA was extracted from each tissue using TriZol^®^ Reagent (Thermo Fisher Scientific) according to the manufacturer’s instructions. The RNA concentration was measured with a NanoDrop ND-1000 spectrophotometer (Thermo Fisher Scientific).

Reverse transcription (RT) was performed with 10 ng of RNA using the High-Capacity cDNA Reverse Transcription Kit^®^ (Thermo Fisher Scientific). Probes for hsa_piR_022710 (5′ TAGA TGGT TCAC ATCA CAGG ACTC TGT GC 3′), hsa_piR_019822 (5′ GCAT TGGT GGTA TAGT GGTA AGCA TA GC 3′), and hsa_piR_020840 (5′ TAAA AAAA TGAT GAGT TCAT GTCC TTTG TA 3′) were used for RT (Thermo Fisher Scientific). Real-time PCR was conducted on a 7500 Real-Time PCR System (Applied Biosystems, Thermo Fisher Scientific) using TaqMan^®^ Universal PCR Master Mix (Thermo Fisher Scientific) following the manufacturer’s instructions. Hsa-miR-191 (Assay ID: 002299; TaqMan MicroRNA Assay; Thermo Fisher Scientific) or RNU6B (Assay ID: 001093; TaqMan MicroRNA Assay; Thermo Fisher Scientific) was used as an endogenous control. PCR conditions were as follows: an initial step at 50 °C for 2 min and a denaturation step at 95 °C for 10 min, followed by 40 cycles of 15 s at 95 °C and 1 min at 60 °C. The relative expression of piRNAs was calculated using the 2^−ΔΔCt^ method, where Ct_piRNA_ − Ct_miR-191_ = ΔCt and ΔCt_sample_ − ΔCt_Calibrator_ (0.63 for hsa_piR_0198022 and 8.86 for hsa_piR_022710) = ΔΔCt.

### 4.3. Statistical Analyses

The last day of follow-up is defined as the last day that the patient attended an appointment for lung cancer follow-up. DFS was calculated from the date of surgery to the first event (relapse, death, or last day of follow-up), OS was defined as the time from surgery to death by any cause or the last day of follow-up, and TTR was measured from surgery to relapse or the last day of follow-up.

The sample size was determined through a combination of a literature review and statistical power analysis using G*Power software (version 3.1.9.7, University of Düsseldorf, Düsseldorf, Germany). An a priori power analysis was conducted to estimate the minimum sample size required to detect statistically significant differences, with an α level set at 0.05 and a power of 90%. The analysis indicated that a minimum of four patients was required for both the tumor tissue and normal tissue groups, thirteen patients for the LUAD group, and nine for the LUSC group. Additionally, sixteen samples per group were needed for recurrence analysis, six samples per group for DFS, and twenty-seven patients per group for OS analysis.

Shapiro–Wilk and Kolmogorov–Smirnov tests were applied to assess data normality. As data were not normally distributed (*p* < 0.001), non-parametric tests were employed. The Mann–Whitney U test was used for two-group comparisons, while the Kruskal–Wallis test was applied for comparisons among three or more groups. Chi-square tests were used for categorical variable analyses. Spearman correlation coefficients were calculated to assess the correlation matrix among piRNAs. Paired *t*-tests and Wilcoxon signed-rank tests were employed to compare piRNA expression levels in tumor and adjacent normal tissues. Differences in expression between LUAD and LUSC subtypes were evaluated using the Mann–Whitney U test. The MetaboAnalystR package was used for calibration and clustering analysis. Hierarchical clustering was performed to visualize the grouping of samples based on piRNA expression levels. To identify prognostic predictors, univariate Cox regression analysis was conducted, followed by multivariate Cox regression for variables with *p* < 0.05 in the univariate analysis. ROC analysis was performed to evaluate the diagnostic performance of individual piRNAs and their combinations, calculating the AUC, sensitivity, and specificity. A CART model was developed to predict recurrence risk based on piRNA expression levels. Patients were stratified into high-, medium-, and low-risk groups based on cutoff values determined using X-tile software [52]. Cross-validation was employed to validate the robustness of the CART model. Calibration plots were constructed to analyze how well the probabilities predicted by the model aligned with the actual observed frequencies in the data. Kaplan–Meier survival curves were generated to estimate the TTR, DFS, and OS of patients with different expression levels of piRNAs. Patients were stratified into high-, medium-, and low-expression groups based on the median expression level of piRNAs, and differences were analyzed using the log-rank test.

All statistical analyses were conducted using IBM SPSS Statistics v29, GraphPad Prism v10.4.1, and R v4.4.2. The level of significance was set at *p* < 0.05. Figures and visualizations were created using R and GraphPad Prism software.

## 5. Conclusions

In summary, we demonstrated that hsa_piR_022710, hsa_piR_019822, and hsa_piR_020840 can help distinguish NSCLC histology at the molecular level and are promising biomarkers for LUSC patients. Higher levels of hsa_piR_022710 and hsa_piR_019822 were associated with increased post-surgery disease recurrence and shorter OS. As our sample size was not large and lacked validation across diverse populations, further prospective studies are needed to confirm these findings as well as their oncogenic mechanisms in NSCLC. We believe hsa_piR_022710, hsa_piR_019822, and hsa_piR_020840 are valuable biomarkers for guiding adjuvant therapy decisions in LUSC patients.

## Figures and Tables

**Table 1:** Patients’ main clinicopathological characteristics.

Characteristics	Total Patients N = 260 (%)	LUAD Patients N = 145 (%)	LUSC Patients N = 97 (%)
**Sex**			
Male	191 (73.5)	89 (61.4)	88 (90.7)
Female	69 (26.5)	56 (38.6)	9 (9.3)
**Age (years), mean (range)**	67 (32–85)	66 (32–85)	68 (50–83)
≤65	109 (41.9)	65 (44.8)	37 (38.1)
>65	151 (58.1)	80 (55.2)	60 (61.9)
**ECOG PS**			
0	65 (25)	35 (24.1)	24 (24.79)
1	188 (72.3)	108 (74.5)	72 (74.2)
2	7 (2.7)	2 (1.4)	1 (1)
**Tumor size (pT)**			
≤30 mm	115 (44.3)	70 (48.3)	39 (40)
31–50 mm	85 (32.7)	44 (30.3)	36 (37)
51–70 mm	43 (16.5)	22 (15.2)	19 (20)
>70 mm	17 (6.5)	9 (6.2)	3 (3.1)
**Lymph node status (pN)**			
0	190 (73.1)	109 (75.2)	68 (70.1)
1	52 (20)	24 (16.6)	23 (23.7)
2	17 (6.5)	11 (7.6)	6 (6.2)
3	1 (0.4)	1 (0.7)	0
**Lymph node status (pN)**			
Negative	190 (73.1)	109 (75.2)	68 (70.1)
Positive	70 (26.9)	36 (24.8)	29 (29.9)
**Stage**			
I	157 (60.4)	91 (62.8)	55 (56.7)
II	57 (21.9)	25 (17.2)	28 (28.9)
III	46 (17.7)	29 (20)	14 (14.4)
**Histology**			
Adenocarcinoma	145 (55.8)		
Squamous cell carcinoma	97 (37.3)		
Others	18 (6.9)		
**Smoking history**			
Current smoker	97 (37.3)	51 (35.2)	39 (40.2)
Former smoker	134 (51.6)	67 (46.2)	58 (59.8)
Non-smoker	29 (11.2)	27 (18.6)	0
**Type of surgery**			
Lobectomy	206 (79.2)	121 (83.4)	72 (74.2)
Pneumonectomy	17 (6.6)	7 (4.8)	9 (9.3)
Bilobectomy	7 (2.7)	2 (1.4)	4 (4.1)
Atypical resection	16 (6.2)	9 (6.2)	5 (5.2)
Typical segmentectomy	14 (5.4)	6 (4.1)	7 (7.2)
**Adjuvant treatment**			
Yes	79 (30.4)	42 (29)	31 (32)
No	181 (69.6)	103 (71)	66 (68)
**Relapse**			
Yes	106 (40.8)	65 (44.8)	32 (33)
No	154 (59.2)	80 (55.2)	65 (67)
**Average follow-up time (months)**	58	65	56

Abbreviations: LUAD, lung adenocarcinoma; LUSC, lung squamous cell carcinoma, ECOG PS, Eastern Cooperative Oncology Group Performance Status.

## Data Availability

The data that support the findings of this study are available from the corresponding author upon reasonable request.

## Supplementary Materials

The following supporting information can be downloaded at https://www.mdpi.com/article/10.3390/ijms26072870/s1.

## Abbreviations

The following abbreviations are used in this manuscript:

piRNAs	PIWI-interacting RNAs
OS	Overall survival
DFS	Disease-free survival
LUSC	Lung squamous cell carcinoma
NSCLC	Non-small-cell lung cancer
LUAD	Lung adenocarcinoma
TTR	Time to Relapse
ROC	Receiver operating characteristic
FBS	Fetal bovine serum
RT	Reverse transcription
