# Supplementary material for: Diagnostic and Prognostic Value of hsa_piR_022710, hsa_piR_019822, and hsa_piR_020840 in Early-Stage Non-Small-Cell Lung Cancer: Implications for Recurrence and Survival in Squamous Cell Carcinoma Patients
Source: Int J Mol Sci. 2025 Mar 21;26(7):2870. doi: 10.3390/ijms26072870 (PMC11989015; doi:10.3390/ijms26072870)
Supplement: Supplementary file 1 [file ijms-26-02870-s001.zip › ijms-3514956-supplementary.pdf]

	Total patients N=260														
Characteristics	TTR-DFS					TTR-Recaida					OS-VM				
	1 year	2 years	3 years	5 years	All time	1 year	2 years	3 years	5 years	All time	1 year	2 years	3 years	5 years	All time
Sex	0.038	0.055	0.116	0.124	0.037	0.034	0.079	0.195	0.219	0.275	0.058	0.035	0.073	0.117	0.012
Age (years),> 65	0.12	0.137	0.186	0.332	0.329	0.128	0.112	0.182	0.297	0.245	0.689	0.774	0.6	0.773	0.586
ECOG PS	0.306	0.467	0.34	0.245	0.205	0.451	0.649	0.586	0.465	0.554	0.166	0.097	0.055	0.221	0.429
Tumor size(cm)	0.004	0.024	0.026	0.146	0.21	0.003	0.01	0.012	0.069	0.046	0.506	0.031	0.073	0.428	0.77
Lymph node status (pN) 1,2,3	0.034	0.002	0.012	0.083	0.046	0.011	<0.001	0.002	0.018	<0.001	0.713	0.003	0.004	0.024	0.006
Lymph node status (pN) +/-	0.011	<0.001	0.003	0.025	0.024	0.003	<0.001	<0.001	0.003	<0.001	0.858	0.011	0.019	0.051	0.044
Stage	0.003	0.001	0.002	<0.001	0.01	<0.001	<0.001	<0.001	<0.001	<0.001	0.55	<0.001	0.001	0.002	0.004
Histology	0.021	0.149	0.272	0.438	0.786	0.011	0.077	0.137	0.286	0.15	0.507	0.668	0.905	0.977	0.449
Smoking history	0.673	0.538	0.647	0.519	0.497	0.615	0.481	0.569	0.541	0.721	0.757	0.082	0.306	0.421	0.226
Type of surgery	0.505	0.537	0.794	0.842	0.901	0.351	0.46	0.578	0.579	0.331	0.828	0.473	0.78	0.829	0.993
Adjuvant treatment	0.049	0.173	0.458	0.654	0.844	0.017	0.085	0.209	0.266	0.277	0.296	0.639	0.886	0.67	0.578
Relapse	<0.001	<0.001	<0.001	<0.001	<0.001						0.04	<0.001	<0.001	<0.001	<0.001
Emphysema	0.397	0.504	0.251	0.219	0.124	0.415	0.535	0.278	0.294	0.496	0.2	0.197	0.123	0.043	0.002

	LUAD N= 145														
Characteristics	TTR-DFS					TTR-Recaida					OS-VM				
	1 year	2 years	3 years	5 years	All time	1 year	2 years	3 years	5 years	All time	1 year	2 years	3 years	5 years	All time
Sex	0.096	0.078	0.157	0.119	0.099	0.096	0.087	0.222	0.168	0.245	0.171	0.115	0.173	0.244	0.127
Age (years), > 65	0.611	0.303	0.462	0.611	0.749	0.611	0.306	0.388	0.556	0.572	0.897	0.979	0.708	0.354	0.319
ECOG PS	0.326	0.506	0.555	0.672	0.716	0.326	0.614	0.605	0.717	0.762	0.777	0.498	0.17	0.32	0.702
Tumor size(cm)	0.004	0.005	0.003	0.032	0.013	0.004	0.001	<0.001	0.008	0.002	0.717	0.171	0.073	0.246	0.273
Lymph node status (pN) 1,2,3	0.066	0.001	0.002	0.003	0.001	0.066	<0.001	<0.001	0.001	<0.001	0.736	0.01	0.005	0.011	0.001
Lymph node status (pN) +/-	0.015	<0.001	<0.001	<0.001	<0.001	0.015	<0.001	<0.001	<0.001	<0.001	0.813	0.125	0.033	0.037	0.005
Stage	0.003	0.004	0.011	0.011	0.027	0.003	<0.001	0.001	0.002	<0.001	0.463	0.013	0.052	0.098	0.03
Smoking history	0.853	0.514	0.387	0.502	0.642	0.853	0.49	0.472	0.594	0.711	0.83	0.096	0.237	0.278	0.334
Type of surgery	0.53	0.319	0.241	0.274	0.258	0.53	0.195	0.119	0.082	0.078	0.827	0.492	0.403	0.277	0.499
Adjuvant treatment	0.015	0.028	0.132	0.147	0.182	0.015	0.01	0.041	0.043	0.014	0.986	0.35	0.457	0.719	0.31
Relapse	<0.001	<0.001	<0.001	<0.001	<0.001						0.148	<0.001	<0.001	<0.001	<0.001
Emphysema	0.304	0.438	0.202	0.245	0.204	0.304	0.533	0.328	0.403	0.434	0.092	0.089	0.021	0.011	0.005

	LUSC N=97														
Characteristics	TTR-DFS					TTR-Recaida					OS-VM				
	1 year	2 years	3 years	5 years	All time	1 year	2 years	3 years	5 years	All time	1 year	2 years	3 years	5 years	All time
Sex	0.166	0.277	0.226	0.249	0.121	0.19	0.358	0.318	0.417	0.435	0.389	0.457	0.404	0.451	0.194
Age (years), > 65	0.047	0.228	0.136	0.253	0.169	0.049	0.151	0.2	0.247	0.175	0.804	0.424	0.143	0.339	0.405
ECOG PS	0.699	0.561	0.233	0.115	0.305	0.812	0.84	0.507	0.375	0.678	0.281	0.115	0.067	0.147	0.268
Tumor size(cm)	0.9	0.906	0.798	0.777	0.544	0.898	0.933	0.948	0.947	0.987	0.403	0.573	0.626	0.763	0.53
Lymph node status (pN) 1,2,3	0.152	0.328	0.681	0.877	0.339	0.079	0.206	0.428	0.854	0.67	0.21	0.14	0.371	0.763	0.29
Lymph node status (pN) +/-	0.292	0.265	0.523	0.728	0.28	0.155	0.185	0.284	0.802	0.639	0.077	0.283	0.669	0.976	0.367

Stage	0.147	0.095	0.202	0.249	0.441	0.06	0.066	0.212	0.229	0.499	0.51	0.07	0.113	0.067	0.23
Smoking history	0.203	0.533	0.521	0.499	0.905	0.456	0.958	0.969	0.35	0.766	0.082	0.767	0.709	0.962	0.805
Type of surgery	0.383	0.923	0.954	0.947	0.894	0.575	0.988	0.995	0.979	0.893	0.821	0.667	0.901	0.714	0.797
Adjuvant treatment	0.87	0.327	0.278	0.116	0.01	0.893	0.369	0.333	0.205	0.106	0.063	0.463	0.369	0.211	0.018
Relapse	<0.001	<0.001	<0.001	<0.001	<0.001						0.026	<0.001	<0.001	<0.001	<0.001
Emphysema	0.709	0.887	0.932	0.636	0.371	0.783	0.956	0.697	0.526	0.768	0.597	0.711	0.767	0.875	0.095
